# Treatment of large proximal ureteral stones: extra corporeal shock wave lithotripsy versus semi-rigid ureteroscope with lithoclast

**DOI:** 10.1186/1755-7682-3-3

**Published:** 2010-01-28

**Authors:** Ehab R Tawfick

**Affiliations:** 1 Urologu Department, El-Minia University, El-Mehata Street (Borg Savoy), El-Minia City, Posta Code 61111, Country Egypt

## Abstract

**Purpose:**

Assessment of safety and efficacy of extracorporeal shockwave lithotripsy versus semi-rigid ureteroscope with lithoclast for treatment of large proximal ureteral stones.

**Materials and methods:**

The study included 147 patients with large upper ureteral stones. SWL and ureteroscopy were performed in 71 and 76 patients respectively. Patients in the SWL group were treated with Siemens: - Modularis lithovario under intravenous sedation on an out patient basis. Patients in the ureteroscopy group were treated with (7.5 Fr) semi-rigid ureteroscope and lithoclast under spinal anesthesia on a day care basis.

**Results:**

Stone - free rate for in situ SWL was 58% (41 of 71) patients. For semi-rigid ureteroscope accessibility of the stones was 94% (72 of 76) and the stone free rate was 92% (70 of 76) No major complications were encountered in both groups.

Mean stone size was 1.34 ± 0.03 cm in the SWL group and 1.51 ± 0.04 in the ureteroscopy group.

**Conclusions:**

Our study demonstrates that ureteroscopy with lithoclast can be considered as acceptable treatment modality for large proximal ureteral calculi and can be considered as fist line for treatment of large proximal ureteral stones.

## Introduction

Most ureteral stones pass spontaneously. Those that do not can be removed by either shock wave lithotripsy or ureteroscopy. Open surgery is appropriate as a salvage procedure or in certain unusual circumstances. SWL has been recommended as first line treatment for proximal ureteral calculi less than I cm. for large proximal ureteral calculi it remains to be defined [1].

Stone size is an important variable in determining the out come of SWL, but little information is available on the influence that stone size has on the treatment of proximal ureteral stones. Advances during the last 2 decades with the advent of small diameter ureteroscopes and intra corporeal lithotripsy such as ultrasound, electro hydraulic, lithoclast and more recently the Holmium: YAG laser, have allowed more successful and safer endoscopic removal of upper ureteral calculi [2-5]. In this study for treatment of large proximal ureteral stones we compared treatment outcomes in patients undergoing semi-rigid ureteroscope and lithoclast with in situ SWL.

## Materials and methods

This study included 147 patients with lager upper ureteral stones (more than 1 cm) treated at urology department El Minia university hospital in the period from June 2001 to November 2007.

Patients were informed about the SWL and ureteroscopy as the two treatment modalities and the advantages and disadvantages and side effects of both techniques were explained to patients. According to patient choice, SWL was performed in 71 patients and urteroscopy in 76 patients.

Pre operatively patients were clinically evaluated by plain X-ray of the kidney, ureter and bladder, ultrasound and or excretory urography to confirm stone size, location and degree of hydronephrosis. The upper ureter was defined as the segment between the ureteropelvic junction and the upper border of the sacroiliac joint. The Inclusion criteria included proximal ureteral stones more than one cm. that fails to pass spontaneously causes recurrent renal colic and or obstructive uropathy. Patients with active urinary tract infection, congenital anomalies and previous SWL, stent placement or open surgery of the ureter were excluded.

Ureteroscopy was preformed using long semi-rigid ureteroscope 7.5 Fr. Pre operative antibiotic was administered, spinal anesthesia was used in most of the patients, cystoscopy was performed and retrograde pyelogram then guide wire (GW) was introduced past the stone, Glide wire was used when required. In case of difficulty to pass the GW it was introduced under vision through the ureteroscope, balloon dilation was used. Lithoclast was used to disintegrate the stone using a 2-3 Fr probe in single or multiple modes the number of shocks could be adjusted to avoid stone migration. A stone cone or nitinol tipless dormia basket was used to guard against stone migration when expected. Significant gravels were retrieved using dormia basket. Double J stent, 5-6 Fr, was placed at the end of the procedure in all except 3 patients. The stent was left for 2-3 weeks based on the degree of impaction of the stone and manipulations performed and were removed on out patient basis. All patients were treated on a day care basis.

Patients with in situ SWL were treated using (Siemens modularis litho vario) lithotriptor under intravenous sedation (Bethidine). The used voltage ranged from 12 to17 K.v. The maximum number of shocks was 3000. At the start the rate of shock wave/minute was adjusted to 60 for the first 500 shock wave then increased to 90 shock wave/minute. Patients were treated on an out patient basis. Post treatment abdominal X ray was obtained 2-3 weeks after SWL. The characteristics of patient age, sex and stone size were determined for each group. Stone analysis was performed using crystallography when possible.

Post operative evaluation included KUB, ultrasound for all patients, occasionally excretory urography or non contrast helical CT until the patient is stone free. Treatment outcomes were assessed by being stone free on KUB I month after treatment. Re-treatment and additional procedures were documented. Statistical comparison between both groups was used by the Fisher 2- sided exact test.

## Results

Ureteroscopy was performed in 76 patients; in 72 patients the stones were accessible, while in 4 patients due to Angulations/tightness of the ureter it was difficult to reach the stone. The initial stone free rate of ureteroscopy using lithoclast was 92%. The procedure failed in 2 patients due to edema and angulations at the site of the stone, both of them were treated by open surgery.

Double J stents were inserted in all successfully treated patients due to large stones and to avoid post operative obstruction and aid in stone passage after removal of the stents. Balloon dilation was used for most of patients to facilitate stone retrieval. Trans ureteroscopic balloon dilation just distal to the stone after disimpaction was done in 3 patients with stricture and edema below the stone.

A stone cone was placed under vision to avoid proximal stone migration in most of the patients after stone disimpaction. In patients with too hard stones a nitinol tipless dormia basket with detachable handle was used to catch the stone before disintegration to achieve good contact of the probe with the stone. The mean operative time was 52 minutes (range 38-98). Ureteral stents were left for 2-3 weeks. Patient's age, sex and stone characteristics were comparable between the 2 groups of patients (table 1).

**Table 1:** Patients & stone characteristics

	No. of patients	Average Age	**Male: female ratio**.	Mean operative time (range minute)	Mean stone size (cm) ± SD	Stone free rate %
**Ureteroscopy group**	76	39	61:15	52 (38-98)	1.51 ± 0.04	92%

**SWL group**	71	42	54:17	68 (59-78)	1.34 ± 0.03	58%

**P value**				0.0924	0.6039	0.0003

SWL was performed in 71 patients. The initial stone - free rate for in situ SWL was 58% (41 of 71) patients. The mean operative time was 68 minutes range (59 - 78). In 13 patients with failed SWL a second SWL session was performed which succeeded in 2 patients. Ureteroscopy was done for 14 patients with failed SWL of whom 12 (86%) became stone free. percutaneous stone management was performed successfully for one patient. The remaining patients preferred to do open surgery. Stone analysis was performed for 23 patients in whom stone fragments were available for analysis (table 2).

**Table 2:** Stone Composition

Stone composition	Ureteroscopy group	SWL GROUP
**COM**	11 (15%)	-

**COD**	26 (37%)	12 (52%)

**Calcium phosphate**	9 (13%)	-

**Uric acid**	11 (15%)	7 (30%)

**Mixed**	13 (18%)	4 (17%)

**COM = **calcium oxalate monohydrate

**COD = **calcium oxalate dihydrate

The results of our study clearly demonstrates that the ureteroscopy group received better results compared to SWL group (p = 0.003). There were no major complications in each group. There were recurrent attacks of renal colic requiring emergency ureteroscopy in 1 case, hematuria and flank soreness in the SWL group. Most of the complaints after ureteroscopy were related to stents.

## Discussion

Shock wave lithotripsy (SWL) is the least Invasive treatment for upper urinary tract calculi and is recommended as first line therapy [1]. Stone clearance after SWL is variable and influenced by stone size, location and composition. The results of treatment for proximal ureteral calculi either in situ or after stent placement range from 57 to 96% with a high re-treatment rate of 5 to 60% [1,6-9].

The success rate of repeat SWL after failed initial SWL treatment is relatively low [10]. SWL has success rate above 80% for small upper ureteral stones. However, the success rate for large impacted upper ureteral calculi is low with the highest success rate Around 60% [11-15].

Shock wave lithotripsy does not assure complete relieve of obstruction and is associated with prolonged attacks of pain during stone passage.

Our success rate for SWL in this study after single session was 58% this is comparable to other studies [8,14,15]. This low success rate could be attributed to the limited number of shock waves in single session and the large size of the stones requiring higher power index [[7,8], and [14]]. It is also important to mention that all cases in our study were treated in situ.

Complications in SWL group included post operative pain (colic) requiring emergency ureteroscopy in 1 patient, haematuria, flank soreness and urosepsis. Re-treatment with SWL for 18 patients succeeded only in 3 patients confirming the low success rate of repeat SWL [10]. The ability to predict the response of a stone to shock wave lithotripy would optimize ureteral stone management [1].

Recent development of small diameter semi-rigid and flexible ureteroscopes with the availability of Holmium YAG laser markedly improved the success rate for treating proximal ureteral stones. A success rate of around 50% for proximal ureteral calculi using large diameter rigid ureteroscopes improved to greater than 90% using small diameter ureteroscopes [14-17].

Most of the studies dealing with ureteroscopy for proximal ureteral stones use the Holmium YAG laser for disintegration [14,15,17] being able to destroy all forms of stones using small diameter quartz fibers, large calculi can be fragmented in to dust like particles during fragmentation decreasing the need for fragment retrieval. It can also be used through rigid and flexible ureteroscopes [15-17]. The only disadvantage of Holmium YAG laser is its cost. In this study we used pneumatic lithotripsy (lithoclast) for disintegration being cost-effective, available, and effective, comes with small diameter probes and can be used through durable semi-rigid ureteroscopes.

Balloon dilation was frequently used because stone fragments are larger with the lithoclast compared to Holmium YAG laser. Significant fragments were retrieved using nitinol tipless dormia basket. Stents were more frequently used due to the same reason large stone size mucosal edema and polyps.

Our initial stone - free rate of ureteroscopic lithoclast lithotripsy for proximal ureteral calculi was 92%, which is lower than but still close to other reported series using the Holmium YAG laser [14,15].

The main difficulty in the ureteroscopy group was failure to approach the stone because of tortuous ureter, angulations and edema at the site of the stone which masks the exposure and disintegration of the calculus. In four patients in our study we were not able to reach the stone due to angulations of the ureter and in 2 more patients even after reaching the stone it was difficult to fragment the stone due to marked edema around the stone. It was helpful to have an adequate irrigation and to negotiate the stone by a second guide wire (under vision) or glide to have good exposure of the stone in impacted cases prior do disintegration.

Proximal migration of the stone is a potential limitation with the use of the lithoclast. Different methods have been used to avoid proximal stone migration including use of combined lithoclast and lithovac, use of Dretler stone cone and ante grade occlusion balloon catheter. In this study stone cone was placed under vision to avoid proximal stone migration, it also helped to sweep the small stone fragments during its removal. For hard stones nitinol tipless dormia basket with detachable handle was used to entrap the stone prior to disintegration. Similar to other studies using stone cone stone migration was avoided [18-20].

Several studies as well as our study proved that treatment out come of ureteroscopy was not influenced by stone burden or composition contrary to SWL results which is influenced by both factors [13-15].

Although ureteroscopy is more invasive than ESWL complications after ureteroscopy were limited in our study which is the same for most recent studies owing to use of small diameter ureteroscope 7 F and effective pneumatic lithotripsy and fine retrieval devices. Most of the complications in our study were related to use of stents [[14-18], and [21]].

Considering the four available methods that can be used for large proximal ureteral calculi (According to the guide lines of American urological association) open surgery, PCN, ureteroscopy and ESW) our study supports the use of ueteroscopy, being effective irrespective of stone size or composition, allows immediate relieve of obstruction, comes with minimal morbidity not affected by obesity, bleeding diathesis or previous open surgery. In addition to safety the economic value of using the durable semi-rigid ureteroscope is attractive.

## Conclusions

In experienced hands use of small diameter semi-rigid ureteroscope and lithoclast with the availability of fine retrieval devices and stone cone allows for safe and effective method for treatment of large proximal ureteral stones. In comparison to SWL it comes with higher stone free rate, comparable complications and ensures immediate relief of obstruction.

## Competing interests

The author declares that they have no competing interests.
